# Treatment Strategies of Colistin Resistance *Acinetobacter baumannii* Infections

**DOI:** 10.3390/antibiotics13050423

**Published:** 2024-05-06

**Authors:** Andria Papazachariou, Renatos-Nikolaos Tziolos, Stamatis Karakonstantis, Petros Ioannou, George Samonis, Diamantis P. Kofteridis

**Affiliations:** 1 Department of Internal Medicine & Infectious Diseases, University General Hospital of Heraklion, 71500 Heraklion, Greece; apapazachariou@hotmail.com (A.P.); r.nikos.tz@gmail.com (R.-N.T.); stamatiskarakonstantis@gmail.com (S.K.);; 2 Metropolitan Hospital, Neon Faliron, 18547 Athens, Greece

**Keywords:** *Acinetobacter baumannii*, colistin, resistance, mechanisms, treatment

## Abstract

*Acinetobacter baumannii* has emerged as a pressing challenge in clinical practice, mainly due to the development of resistance to multiple antibiotics, including colistin, one of the last-resort treatments. This review highlights all the possible mechanisms of colistin resistance and the genetic basis contributing to this resistance, such as modifications to lipopolysaccharide or lipid A structures, alterations in outer membrane permeability via porins and heteroresistance. In light of this escalating threat, the review also evaluates available treatment options. The development of new antibiotics (cefiderocol, sulbactam/durlobactam) although not available everywhere, and the use of various combinations and synergistic drug combinations (including two or more of the following: a polymyxin, ampicillin/sulbactam, carbapenems, fosfomycin, tigecycline/minocycline, a rifamycin, and aminoglycosides) are discussed in the context of overcoming colistin resistance of *A. baumannii* infections. Although most studied combinations are polymyxin-based combinations, non-polymyxin-based combinations have been emerging as promising options. However, clinical data remain limited and continued investigation is essential to determine optimal therapeutic strategies against colistin-resistant *A. baumannii*.

## 1. Introduction

*Acinetobacter baumannii* infections pose a significant threat to human health, particularly within current healthcare settings. This opportunistic pathogen is one of the major causes of nosocomial infections, such as ventilator-associated pneumonia, septicemia, meningitis, wound and urinary tract infections, contributing to substantial morbidity and mortality [1]. The antibiotics that are usually effective against *A. baumannii* infections include carbapenems, polymyxins E and B, sulbactam, piperacillin/tazobactam, tigecycline and aminoglycosides [2]. Yet, multidrug-resistant strains (MDR) have become increasingly prevalent, limiting the effectiveness of conventional antibiotic therapies [3].

Antimicrobial resistance (AMR) has emerged as a globally chronic public health problem, with the forecast of 10 million deaths per year by 2050 [4]. The treatment of *A. baumannii* infections presents a formidable challenge due to its propensity for antimicrobial resistance. In contrast to Enterobacterales for which there are many options against carbapenem-resistant strains (ceftazididime/avibactam ± aztreonam, meropenem/vaborbactam, imipenem/relebactam), new β-lactam/β-lactamase combinations are not active against *A. baumannii*’s OXA carbapenemases [4]. This has sparked interest in the revival of old antibiotics, predominantly colistin [5], which has resulted in an escalating incidence of colistin resistance in *A. baumannii* [6,7], its prevalence being as high as 85% in Greece [8]. The ability of *A. baumannii* to acquire and disseminate resistance mechanisms has profound implications for human health since resistance to last-resort antibiotics, including colistin, has resulted in the emergence of pan-drug-resistant (PDR) strains [5], which are associated with increased mortality [3] and very limited treatment options [6,7] where new antibiotics (cefiderocol, sulbactam/durlobactam) are not yet available. Of interest is that colistin can still be used as part of combination regimens against colistin-resistant strains as it can work synergistically with other antibiotics even at subinhibitory concentrations [6,7]. Furthermore, various non-polymyxin-based combination regimens have been tried [7]. Notably, even though colistin represents a last resort treatment option for carbapenem-resistant *A. baumannii*, there is conflicting evidence on the impact of colistin resistance on clinical outcomes ranging from lower to higher mortality associated with colistin resistance [8,9,10].

Colistin belongs to the class of polymyxin antibiotics; possessing a positively charged L-diaminobutyric acid, that forms electrostatic bonds with the negatively charged phosphate groups of lipid A, a vital constituent of lipopolysaccharide (LPS) found in Gram-negative bacilli [11]. This interaction is pivotal as lipid A governs bacterial permeability and external exchange [12]. By competitively displacing divalent cations like calcium (Ca^2+^) and magnesium (Mg^2+^), colistin disrupts the structure of LPS, subsequently incorporating its hydrophobic acyl chain. Consequently, the external outer membrane (OM) undergoes expansion, leading to permeabilization and facilitating colistin entry. This process elucidates the synergistic effect observed when colistin is combined with other hydrophilic antimicrobials like β-lactams, gentamicin, rifampicin, meropenem, and tigecycline [13]. In essence, colistin acts by solubilizing the bacterial cell membrane, culminating in a bactericidal outcome [14].

This review aims to provide comprehensive insight into the colistin resistance mechanism in *A. baumannii* and summarize available treatment strategies. By elucidating the molecular mechanisms driving colistin resistance and assessing the effectiveness of different therapeutic approaches, this study aims to contribute to the development of more targeted and efficacious treatments for *A. baumannii* infections.

## 2. Mechanisms of Colistin Resistance

Colistin (polymyxin E) belongs to the class of polymyxin antibiotics; its primary target is the outer bacterial cell membrane and is considered bactericidal [14]. As a cationic polypeptide, colistin interacts with the cell membrane’s negatively charged LPS molecules through the displacement of positively charged ions (Mg^2+^, Ca^2+^), destroying the integrity of the cell membrane [13].

A well-described mechanism of resistance to colistin in *A. baumannii* involves the complete loss of LPS structure due to mutations in or disruption of the LPS biosynthesis genes (*lpxA*, *lpxC*, or *lpxD*). Since colistin primarily targets the lipid A component of LPS, the absence of LPS or alterations in its structure can result in reduced susceptibility or resistance to colistin [15]. The initial study that observed this resistance mechanism involved 13 independent colistin-resistant derivatives of the *A. baumannii* type strain ATCC 19606. Various genetic alterations (nucleotide substitutions, deletions, and insertions) were observed in one of these three genes, ultimately causing frameshifts or truncated proteins impairing lipid A biosynthesis. It is noteworthy that although the mutations found varied in scale, ranging from single nucleotide mutations to large deletions spanning up to 445 nucleotides, they all led to the same outcome: disruption of the synthesis pathway of lipid A, which in turn led to the absence of or reduction in LPS production [16]. In subsequent research, the insertion of *IS Aba1* or *IS Aba11* within the *lpxC* gene has been identified as a frequent occurrence in colistin-resistant *A. baumannii*. This mutation causes the inactivation of the *lpxC* and *lpxA* genes, leading to the loss of LPS. Across different studies, the disruption of the *lpxC* gene has consistently been observed within the same region (321–420 nucleotides), leading to the proposal that this region may serve as a preferred site for the integration of insertion sequences (ISs) [17,18,19,20]. Furthermore, some colistin-resistant *A. baumannii* isolates have shown downregulation of *lpxACD* expression, resulting in reduced LPS production [21]. It should be emphasized that certain amino acid substitutions, such as N287D in *lpxC* and E117K in *lpxD*, have been identified in both colistin-susceptible and colistin-resistant isolates. However, when these substitutions are combined with a mutation in the *pmrCAB* operon, they may exhibit a synergistic effect, contributing to colistin resistance [22,23,24].

Another significant resistance mechanism involves modifying the lipid A fraction of the LPS in the bacterial cell membrane. This modification includes the addition of molecules such as 4-amino-4-deoxy-l-arabinose (L-Ara4N), phosphoethanolamine (PEtN), or galactosamine to lipid A. These alterations in the structure of lipid A lead to a decrease in the net negative charge of the cell membrane. Consequently, this affects the binding of positively charged colistin to the membrane, reducing its efficacy [25,26]. Although the modification of LPS with L-Ara4N has been identified as a common and effective mechanism of colistin resistance in diverse Gram-negative pathogens such as *Salmonella enterica*, *Klebsiella pneumoniae*, *Escherichia coli*, and *Pseudomonas aeruginosa*, this modification was found to be absent in *A. baumannii* [27]. Chin et al. revealed that mutations in *pmrB* can lead to heightened expression of the *naxD* gene. *NaxD* is responsible for modifying lipid A by adding galactosamine, thereby reducing the affinity for cationic colistin and contributing to resistance in *A. baumannii* strains [28]. The addition of PEtN to the 4′-phosphate or 1-phosphate group of lipid A in colistin-resistant *A. baumannii* is primarily mediated by the enzymatic activity of pEtN transferases encoded by *pmrC* [29]. This mechanism is mediated by mutations in genes encoding the *PmrAB* two-component system, leading to overexpression of the phosphoethanolamine transferase *PmrC* [30,31,32,33]. A systematic review found that most in vivo studies highlighted mutations within the *pmrC* AB locus, with *pmrB* mutations being the most prevalent, leading to the upregulation of *pmr* genes. Conversely, mutations in genes crucial for the synthesis of the lipid A component of LPS were more frequently observed in vitro, with only one instance identified in vivo [34]. A study involving the isolation of three colistin-resistant clinical *A. baumannii* strains from distinct patients unveiled that although non-synonymous mutations were present across all domains of *PmrB*, a significant proportion was concentrated within the histidine kinase A (HisKA) domain. This domain is crucial in facilitating autophosphorylation and transferring the phosphoryl group to the *PmrA* response regulator. *PmrA*, upon phosphorylation, serves as a transcriptional regulator capable of modulating the expression of various genes involved in LPS structural modifications, such as the addition of PEtN [35]. In addition, mutations in the receiver domain (REC) of the *PmrA* have been reported in *A. baumannii* strains resistant to colistin [23]. Furthermore, another study investigating colistin resistance in four isogenic isolate pairs of *A. baumannii* reported that *PmrB* mutations led to approximately 10–13 times higher expression of *pmrC* compared to susceptible isolates. However, it is worth mentioning that PTeN addition was also observed in susceptible isolated according to a study investigating indicating that this resistance mechanism might be strain-specific and that PEtN addition alone is not the sole factor leading to colistin resistance [29]. Except for the overexpression of *PmrC*, a mechanism of colistin resistance in *A. baumannii* was observed in some isolates and to a lesser extent the upregulation of the *pmrA* and *pmrB* [36,37,38]. While this observation is anticipated, given that these genes belong to the same operon as the *pmrC* gene (*pmrCAB*), there are instances where no correlation was observed between *PmrAB* and *PmrC* overexpression [39]. It is worth noting that, according to Beceiro et al. who analyzed *PmrCAB* in a diverse array of clinical isolates and laboratory mutants of *A. baumannii*, it was suggested that resistance to colistin necessitates at least two separate genetic occurrences: (i) the occurrence of at least one amino acid alteration in *PmrB* and (ii) the upregulation of *pmrA* and *pmrB* expression [33].

An alternative mechanism documented involving the addition of PEtN that consequently leads to colistin resistance in *A. baumannii* is the overexpression of another *pmrC* homolog, *eptA* (ethanolamine phosphotransferase A). While the sole presence of the *eptA* gene in the bacterial genome does not inherently result in colistin resistance, the insertion of the ISAbal sequence into the DNA preceding the *eptA* gene can enhance the expression of this enzyme. The activity of the *pmrC*-homolog *eptA* results in the modification of lipid A through the addition of PEtN [40]. In a recent investigation on *A. baumannii* isolates collected from a patient before and after ineffective colistin therapy, genomic sequencing of the post-treatment isolate uncovered an additional instance of ISAba125 within the *H-NS* gene, which encodes a transcriptional regulator. As *H-NS* regulates the expression of genes implicated in lipid A modification, including *eptA*, the presence of this extra copy was linked to *H-NS* dysfunction, thereby contributing to colistin resistance [41].

The presence of mcr genes, which encode phosphoethanolamine transferases (MCR enzymes) catalyzing the attachment of phosphoethanolamine to lipid A of LPS, represents a well-known mechanism of colistin resistance in Gram-negative bacteria [42,43]. Until now, bacteria isolated from animals, food, humans, and the environment have revealed the existence of ten distinct *mcr* gene families (*mcr-1* to *mcr-10*) [44]. In the context of *A. baumannii* strains resistant to colistin, it was found that the resistance was primarily caused by specific plasmid-mediated *mrc* genes, such as *mcr-1* and *mcr-4.3* [45,46].

In addition to modifications or loss of LPS structure, *A. baumannii* can develop resistance to colistin through a different mechanism, such as alterations in outer membrane permeability. The outer membrane permeability of *A. baumannii* is notably lower, accounting for less than 5% compared to other Gram-negative bacilli. This is attributed to the relatively small number and size of porins present [47]. According to the literature, colistin resistance in *A. baumannii* mutants has been associated with either the loss or reduced expression of the OmpW porin [48]. Furthermore, specific non-lpx (lipoprotein) proteins involved in the structure and integrity of the outer membrane have been observed to contribute to colistin resistance. This hypothesis emerged from analyzing *A. baumannii* strains exposed to escalating colistin concentrations. The findings indicated that strains lacking LpsB, a non-Lpx protein known as a glycosyltransferase responsible for LPS core synthesis, exhibited heightened susceptibility to colistin. This underscores the potential role of LpsB in facilitating colistin resistance in *A. baumannii* by bolstering the stability of the outer membrane [49]. In addition to the absence of lpsB, single mutations in genes such as *H128Y* and *241K have been documented in *A. baumannii* strains exhibiting colistin resistance [50]. Colistin resistance also correlates with amino acid substitutions in VacJ and PldA non-lpx proteins. PldA, crucial for preserving lipid asymmetry in the outer membrane, and VacJ, a component of the ABC transporter system, undergo mutations that disrupt outer membrane organization, thereby fostering resistance development [51].

Heteroresistance represents a lesser-explored and less understood concept, where a minor fraction of bacterial cells within a clonal population display resistance to an antibiotic. At the same time, the majority remain susceptible [50,51]. Li et al. were the first to define colistin heteroresistance in *A. baumannii* as the occurrence of resistance to colistin within a subpopulation despite the majority being susceptible with a minimum inhibitory concentration (MIC) of ≤2 mg/L [52,53]. Prior administration of colistin may pose a risk for an increased incidence of heteroresistance [54]. The identification of colistin-heteroresistant *A. baumannii* strains in clinical samples serves as a significant cautionary signal that inappropriate colistin usage could lead to rapid resistance development and treatment ineffectiveness [55].

Table 1 summarizes all the abovementioned mechanisms of resistance.

## 3. Available Treatments of *A. baumannii* Colistin Resistance

The approach to treating colistin-resistant *A. baumannii* is similar to the approach for carbapenem-resistant *A. baumannii* [56,57], with the main difference being that colistin is not appropriate as a monotherapy and may not be the preferable backbone for combination therapy. Of note colistin resistance in carbapenem-resistant *A. baumannii* strains may result in pandrug resistance where new antibiotics (cefiderocol and sulbactam/durlobactam) are not available.

Evidence on the treatment of carbapenem-resistant *A. baumannii* is limited due to the observational (and predominantly retrospective) design of the majority of available studies, with multiple limitations (including heterogeneous patient populations, heterogeneous *A. baumannii* susceptibilities and mechanisms of resistance, heterogeneous treatment regimens, and small study populations). Multiple meta-analyses [58,59,60,61] (including network meta-analyses [62,63,64]) have been conducted to determine the optimal treatment regimen against carbapenem-resistant *A. baumannii*, but the results of these studies are difficult to interpret and limited by the quality of available studies. Notably, the majority of studies have been conducted in patients with infections by colistin-susceptible *A. baumannii* and most studied combinations are polymyxin-based. Guidelines have been published by both the Infectious Diseases Society of America (IDSA) [56] and the European Society of Clinical Microbiology and Infectious Diseases (ESCMID) [65]; however, these were published before approval of sulbactam/durlobactam (which now represent a first-line treatment option where available) and recommendations against cefiderocol may change considering subsequent encouraging real-life data [57].

Unfortunately, cefiderocol and sulbactam/durlobactam are not yet widely available. Furthermore, sulbactam/durlobactam is not active against metallo-β-lactamase producing *A. baumannii*, which, in combination with the high prevalence of cefiderocol non-susceptibility in these strains [66,67], could prove to be a problem in the future. As these new antibiotics are increasingly being used in clinical practice, there is a risk of selection and spread of New Dehli Metallo-beta-lactamase (NDM)-producing *A. baumannii*, which is already increasingly being reported [68,69]. This leaves synergistic antibiotic combinations as the only treatment option against colistin-resistant (typically PDR) *A. baumannii* strains [6,7]. Multiple antimicrobial combination regimens have been evaluated and are being used in clinical practice, but the optimal regimen remains unclear [7]. Current guidelines recommend a sulbactam-based (ampicillin/sulbactam or sulbactam/durlobactam if the latter is available) treatment regimen. Sulbactam can be combined with several antibiotics, predominantly, colistin, tigecycline and cefiderocol [56,57]. The various treatment options and considerations for selecting a treatment regimen are summarized in Table 2 and Table 3.

## 4. Conclusions

In understanding the mechanisms of colistin resistance in *A. baumannii*, it becomes evident that the bacterium employs a multifaceted approach to evade the antimicrobial effects of this last-resort antibiotic. Primarily, alterations in the lipid A component of the LPS structure play a pivotal role. Adding molecules like *PEtN*, or galactosamine to lipid A alters its structure, reducing the affinity for colistin. Furthermore, mutations or disruptions in LPS biosynthesis genes, such as *lpxA*, *lpxC*, or *lpxD*, lead to the loss or modification of LPS, rendering the bacterium less susceptible to colistin. Additionally, mutations affecting outer membrane permeability, such as the loss or reduced expression of porins or mutations in non-Lpx proteins contribute to colistin resistance. Heteroresistance further complicates treatment, potentially leading to failure if not addressed appropriately.

Despite colistin being a last resort treatment option for infections by carbapenem-resistant *A. baumannii*, resistance to colistin likely does not pose a major problem yet given the availability of several non-polymyxin-based regimens as well as the potential to still use colistin as part of synergistic combination regimens. The currently preferred backbone for the treatment of carbapenem-resistant colistin-resistant *A. baumannii* is sulbactam (sulbactam/durlobactam if available, or else ampicillin/sulbactam). However, the optimal combination regimen remains unclear.

## Figures and Tables

**Table 1:** Summary of the mechanisms of resistance to colistin in *A. baumannii*.

Mechanism of Colistin Resistance
**Modification of LPS**
Mutations leading to deficient or complete loss of LPS structure
Mutations in *lpxACD* genes leading to decreased LPS production
**Addition of positively charged molecules**
Addition of galactosamine to lipid A fraction of LPS through mutations in *pmrB*
Overexpression of *pmrC* leads to the modification of lipid A fraction of LPS through the addition of pEtN
Overexpression of *EptA* leads to modification of lipid A fraction of LPS through the addition of pEtN
Dysfunction of *H-NS*
**Horizontal gene transfer**
Horizontal gene transfer enables the acquisition of mcr genes, encoding *pEtN* transferases that attach *pEtN* to lipid A
**Alterations in outer membrane permeability**
Overexpression of efflux pumps (EmrAB)–Decreased expression of OmpW porin
Expression of specific non-lpx proteins involved in the structure and integrity of the outer membrane
**Heteroresistance**
Occurrence of resistance within a subpopulation through spontaneous mutations or the presence of resistance genes despite majority susceptibility, leading to rapid resistance development.

Abbreviations: LPS: lipopolysaccharide, pEtN: phosphoethanolamine, lpx: lipoprotein.

**Table 2:** Summary of treatment options for colistin-resistant *A. baumannii*.

Antibiotic	Comment
Sulbactam/durlobactam	The preferred treatment option where available [57]. Retains activity against most XDR/PDR *A. baumannii* strains [70], including strains resistant to colistin and cefiderocol [71]. Has shown non-inferiority and lower nephrotoxicity compared to colistin in the registrational phase 3 trial [72]. **Limitations:** Limited clinical evidence Sulbactam/durlobactam was used in combination with imipenem/cilastatin (to cover other co-infecting pathogens) in the registrational phase 3 trial [72]. At least based on in vitro data the impact of imipenem on the activity of sulbactam/durlobactam appears to be minimal [70]. Not active against metallo-β-lactamase-producing *A. baumannii* Not yet widely available
Cefiderocol	Retains activity against most XDR/PDR *A. baumannii* strains [66]. Despite initial disappointing data from subgroup analyses from the randomized trials [73,74], subsequent studies and meta-analyses are more encouraging [57,58,59]. **Limitations:** Not yet widely available In available randomized trials cefiderocol performed worse than the best available therapy (CREDIBLE [73]) and similar to high-dose meropenem (APEKS-NP [74]), which is inactive alone, in subgroup analyses of patients with *A. baumannii* infections. Subsequent real-life studies are promising but observational and mostly retrospective, and with mixed results [57,58,59]. Conflicting breakpoints comparing EUCAST and CLSI [66] High prevalence of resistance/heteroresistance in some settings [66] Especially high prevalence of resistance and risk of treatment-emergent resistance in metallo-β-lactamase-producing strains [66]
Ampicillin/sulbactam	Sulbactam is the active component. Used in high doses: 27 g/day (18 g ampicillin/3 g sulbactam) infused over 24 h or divided into three doses and infused over 4 h. Most XDR *A. baumannii* strains have a sulbactam MIC of 32–64 mg/L, and only a small proportion have an MIC below the CLSI-defined breakpoint for ampicillin/sulbactam [71,75]. Based on PK/PD data high-dose sulbactam may cover isolates with MICs as high as 32–64 mg/dL [76]. **Limitations:** Breakpoints for sulbactam have not been established yet. Breakpoints for ampicillin/sulbactam underestimate the treatment potential considering that the doses used for *A. baumannii* are likely sufficient for sulbactam MICs as high as 32–64 mg/dL [76], and maybe even higher in synergistic combination regimens. Ampicillin/sulbactam is still recommended even in case of resistance to ampicillin/sulbactam, as defined based on CLSI breakpoints [56]. Optimal dosing regimen and adjustment for kidney function not yet clear.
Colistin/Polymyxin-B	Even in case of resistance, it can be useful as part of synergistic combination regimens (subinhibitory concentrations are sufficient for synergy) [6,7]. With the exception of urinary tract infections, polymyxin-B is preferable to colistin where available due to better pharmacokinetic properties and lower risk of nephrotoxicity. Despite conflicting recommendations by guidelines [56,77], nebulized colistin may be a useful option when administered properly (correct nebulization technique and at high doses) [78,79,80], but larger well-designed studies are necessary to confirm potential benefits. Notably, nebulized colistin may achieve concentrations well above MICs [78], suggesting potential benefit even against colistin-resistant isolates. **Limitations:** Poor penetration in epithelial lining fluid (but promising potential for nebulized colistin). Nephrotoxicity. Dosing adjustment for renal function can be complicated for colistin [77], especially in patients with unstable renal function.
Tigecycline	Good penetration in skin and soft tissue infections and osteoarticular infections. Recommended dose: 200 mg loading dose followed by 100 mg twice daily. **Limitations:** Breakpoints not established Nausea/vomiting/abdominal pain are common side effects and may lead to treatment discontinuation Thrombocytopenia and hypofibrinogenemia are common with high doses used for *A. baumannii* Poor concentration in blood and urine Treatment with tigecycline has been associated with worse outcomes in early studies using lower doses, a finding that has not been confirmed in subsequent studies [61,81], although microbiological cure may be lower [61,81].
Minocycline	Good penetration skin and soft tissue infections and osteoarticular infections. Recommended dosing for *A. baumannii* is 200 mg twice daily. Can also be given orally. **Limitations:** Breakpoints not established [82] Nausea common
Eravacycline	More potent (lower minimum inhibitory concentrations) than tigecycline/minocycline [83]. Can be administered orally. **Limitations:** Breakpoints not established Very limited clinical evidence, predominantly from small case series and small observational studies [83,84,85,86] and potentially poor outcomes in bloodstream infections [83]
Fosfomycin	*A. baumannii* is considered inherently resistant to fosfomycin. However, fosfomycin can be useful as part of synergistic combination regimens based on in vitro data, as well as limited clinical data [7,87,88,89,90]. **Limitations:** Available clinical data have severe limitations. The single randomized trial was small and assessed the combination colistin + fosfomycin vs. colistin monotherapy) in colistin-susceptible isolates [88]. The two other studies are both observational, one prospective [90] and one retrospective [89]. Most isolates in the prospective study were colistin-susceptible [90], while all isolates in the retrospective study were pandrug-resistant [89]. In both studies, very heterogeneous treatment regimens were used. Furthermore, the latest study was very small [89]. Lastly, ampicillin/sulbactam, the currently preferred backbone, was used in very few patients [89,90]. The optimal dosing regimen for fosfomycin when used as part of a combination is unclear.
Ceftazidime/avibactam	Ceftazidime/avibactam is not active against carbapenem-resistant *A. baumannii*. However, where sulbactam/durlobactam is not available, the combination of ceftazidime/avibactam with ampicillin/sulbactam is promising [91,92]. The rationale is that avibactam (similar to durlobactam) may help restore the activity of sulbactam [91,92]. **Limitations:** Virtually no published clinical data on the combination.
Trimethoprim/sulfamethoxazole	Active against some XDR *A. baumannii* strains **Limitations:** Very limited clinical evidence, usually in combination with other antibiotics [93,94]
Aminoglycosides	Active against some XDR *A. baumannii* strains **Limitations:** Very limited clinical evidence Not recommended as monotherapy beyond uncomplicated urinary tract infections
Rifabutin	Using culture media more relevant to in-vivo conditions rifabutin is much more potent than rifampicin [95], and is active even against PDR *A. baumannii* strains [96], and has been shown to be effective in vivo [95]. Rifabutin also demonstrates synergy with polymyxins and the combination may be associated with lower risk of emergent resistance [97]. **Limitations:** Not yet recommended given lack of clinical evidence Currently only available orally (intravenous preparation under development [98,99]) Risk of emergence of resistance during treatment. Topic reviewed by Phillips MC et al. [100]
Azithromycin	Similar to rifabutin, traditional culture media may underestimate azithromycin’s potential. In vitro and in vivo data suggest potential to treat *A. baumannii* with azithromycin, as well as synergy potential with polymyxins [101,102,103]. **Limitations:** Not yet recommended given lack of clinical evidence. Very limited clinical data, all for *P. aeruginosa* [104].
Combination vs. monotherapy	Based on in vitro and in vivo synergy and limitations of monotherapy options guidelines currently suggest combination therapy over monotherapy, especially for severe infections by *A. baumannii*. Where sulbactam/durlobactam is not yet available, guidelines suggest ampicillin/sulbactam as the preferred backbone for combination therapy [56,57]. **Limitations:** Clinical evidence is predominantly based on observational, mainly retrospective studies, and anecdotal experience. Methodology for assessing antimicrobial combinations in vitro needs re-evaluation [105]. There is lack of clinical evidence correlating in vitro synergy with clinical outcomes. **Combinations not to use:** Colistin/meropenem has not shown benefit over colistin monotherapy in randomized clinical trials and meta-analyses [106,107]. There may be potential for triple combinations (e.g., colistin/meronem/ampicillin/sulbactam or colistin/meropenem/fosfomycin) [7,57]. However, given lack of clinical data such combinations cannot be recommended. Colistin/rifampicin has not shown clinical benefit in randomized trials [108,109,110]. As discussed above, colistin/rifabutin may be a better combination, pending clinical validation and availability of an intravenous formulation.

A common limitation to all above options is the lack of strong clinical evidence to guide optimal treatment. Where ampicillin/durlobactam is not available, ampicillin/sulbactam is considered the preferred backbone for combination therapy. Abbreviations: XDR: extensively drug-resistant, PDR: pandrug-resistant, MIC: minimum inhibitory concentration, PK/PD: pharmacokinetic/pharmacodynamic

**Table 3:** Summary of considerations for selecting a treatment regimen for *A. baumannii*.

Consideration	Comment
Infection vs. colonization	Isolation of *A. baumannii* from a non-sterile site does not prove infection. This is especially problematic when *A. baumannii* is isolated from the respiratory tract, urine or ulcers/burns/surgical site as well as in polymicrobial cultures (which are quite common [111]). To differentiate infection vs. colonization it is useful to consider a response to empirical treatment and the patient’s status at the time of *A. baumannii* isolation (in a patient already improving or asymptomatic at the time of *A. baumannii* identification, treatment escalation to better cover *A. baumannii* is unnecessary).
Severity of the infection and comorbidities	For high-risk patients and/or severe infections a more aggressive treatment approach is reasonable (e.g., combination therapy vs. monotherapy).
Antibiotic susceptibility and mechanisms of resistance	Ideally, the treatment regimen should include at least one active antibiotic (if available). If this is not an option then selecting an antibiotic with an MIC close to breakpoints (e.g., tigecycline) is reasonable. Note also that for many of the above-discussed options, breakpoints are not well-established or need revising (see Table 2). Furthermore, appropriate breakpoints for synergistic antibiotic combinations are unclear. In vitro evaluation of synergy combined with PK/PD modeling may be helpful in selecting an appropriate antibiotic combination [112,113,114,115].
Site of infection	Antibiotic selection should take into account the penetration of different options at the site of infection. For example, tigecycline/minocycline have great penetration in soft tissues/bone but low urinary excretion and low. Colistin on the other hand does not achieve sufficient concentration in the epithelial lining fluid.
Adverse effects/history of allergies	Patients often do not tolerate treatment due to adverse effects and/or history of adverse reactions.
Response to treatment	In patients not responding or with recurrent infections it may be reasonable to add a third antibiotic to the treatment regimen [57].

Abbreviations: MIC = minimum inhibitory concentration, PK/PD: pharmacokinetic/pharmacodynamic.

## References

[1] Ayoub Moubareck C., Hammoudi Halat D. (2020). Insights into *Acinetobacter baumannii*: A Review of Microbiological, Virulence, and Resistance Traits in a Threatening Nosocomial Pathogen. Antibiotics.

[2] Michalopoulos A., Falagas M.E. (2010). Treatment of *Acinetobacter* Infections. Expert Opin. Pharmacother..

[3] Karakonstantis S., Gikas A., Astrinaki E., Kritsotakis E.I. (2020). Excess Mortality Due to Pandrug-Resistant *Acinetobacter baumannii* Infections in Hospitalized Patients. J. Hosp. Infect..

[4] Tang K.W.K., Millar B.C., Moore J.E. (2023). Antimicrobial Resistance (AMR). Br. J. Biomed. Sci..

[5] Karakonstantis S., Kritsotakis E.I., Gikas A. (2019). Pandrug-Resistant Gram-Negative Bacteria: A Systematic Review of Current Epidemiology, Prognosis and Treatment Options. J. Antimicrob. Chemother..

[6] Karakonstantis S., Kritsotakis E.I., Gikas A. (2020). Treatment Options for *K. pneumoniae*, *P. aeruginosa* and *A. baumannii* Co-Resistant to Carbapenems, Aminoglycosides, Polymyxins and Tigecycline: An Approach Based on the Mechanisms of Resistance to Carbapenems. Infection.

[7] Karakonstantis S., Ioannou P., Samonis G., Kofteridis D.P. (2021). Systematic Review of Antimicrobial Combination Options for Pandrug-Resistant *Acinetobacter baumannii*. Antibiotics.

[8] Balkan I.I., Alkan M., Aygün G., Kuşkucu M., Ankaralı H., Karagöz A., Şen S., Arsu H.Y., Biçer M., Kaya S.Y. (2021). Colistin Resistance Increases 28-Day Mortality in Bloodstream Infections Due to Carbapenem-Resistant Klebsiella Pneumoniae. Eur. J. Clin. Microbiol. Infect. Dis..

[9] Mantzarlis K., Makris D., Zakynthinos E. (2020). Risk Factors for the First Episode of *Acinetobacter baumannii* Resistant to Colistin Infection and Outcome in Critically Ill Patients. J. Med. Microbiol..

[10] Dickstein Y., Lellouche J., Ben Dalak Amar M., Schwartz D., Nutman A., Daitch V., Yahav D., Leibovici L., Skiada A., Antoniadou A. (2019). Treatment Outcomes of Colistin- and Carbapenem-Resistant *Acinetobacter baumannii* Infections: An Exploratory Subgroup Analysis of a Randomized Clinical Trial. Clin. Infect. Dis..

[11] Deris Z.Z., Akter J., Sivanesan S., Roberts K.D., Thompson P.E., Nation R.L., Li J., Velkov T. (2014). A Secondary Mode of Action of Polymyxins against Gram-Negative Bacteria Involves the Inhibition of NADH-Quinone Oxidoreductase Activity. J. Antibiot..

[12] Velkov T., Thompson P.E., Nation R.L., Li J. (2010). Structure−Activity Relationships of Polymyxin Antibiotics. J. Med. Chem..

[13] Bolla J.-M., Alibert-Franco S., Handzlik J., Chevalier J., Mahamoud A., Boyer G., Kieć-Kononowicz K., Pagès J.-M. (2011). Strategies for Bypassing the Membrane Barrier in Multidrug Resistant Gram-negative Bacteria. FEBS Lett..

[14] Mohapatra S.S., Dwibedy S.K., Padhy I. (2021). Polymyxins, the Last-Resort Antibiotics: Mode of Action, Resistance Emergence, and Potential Solutions. J. Biosci..

[15] Espinal P., Pantel A., Rolo D., Marti S., López-Rojas R., Smani Y., Pachón J., Vila J., Lavigne J.-P. (2019). Relationship between Different Resistance Mechanisms and Virulence in *Acinetobacter baumannii*. Microb. Drug Resist..

[16] Moffatt J.H., Harper M., Harrison P., Hale J.D.F., Vinogradov E., Seemann T., Henry R., Crane B., St. Michael F., Cox A.D. (2010). Colistin Resistance in *Acinetobacter baumannii* Is Mediated by Complete Loss of Lipopolysaccharide Production. Antimicrob. Agents Chemother..

[17] Lim T.P., Ong R.T.-H., Hon P.-Y., Hawkey J., Holt K.E., Koh T.H., Leong M.L.-N., Teo J.Q.-M., Tan T.Y., Ng M.M.-L. (2015). Multiple Genetic Mutations Associated with Polymyxin Resistance in *Acinetobacter baumannii*. Antimicrob. Agents Chemother..

[18] Boinett C.J., Cain A.K., Hawkey J., Do Hoang N.T., Khanh N.N.T., Thanh D.P., Dordel J., Campbell J.I., Lan N.P.H., Mayho M. (2019). Clinical and Laboratory-Induced Colistin-Resistance Mechanisms in *Acinetobacter baumannii*. Microb. Genom..

[19] Mu X., Wang N., Li X., Shi K., Zhou Z., Yu Y., Hua X. (2016). The Effect of Colistin Resistance-Associated Mutations on the Fitness of *Acinetobacter baumannii*. Front. Microbiol..

[20] Moffatt J.H., Harper M., Adler B., Nation R.L., Li J., Boyce J.D. (2011). Insertion Sequence IS *Aba11* Is Involved in Colistin Resistance and Loss of Lipopolysaccharide in *Acinetobacter baumannii*. Antimicrob. Agents Chemother..

[21] Kamoshida G., Yamada N., Nakamura T., Yamaguchi D., Kai D., Yamashita M., Hayashi C., Kanda N., Sakaguchi M., Morimoto H. (2022). Preferential Selection of Low-Frequency, Lipopolysaccharide-Modified, Colistin-Resistant Mutants with a Combination of Antimicrobials in *Acinetobacter baumannii*. Microbiol. Spectr..

[22] Ušjak D., Novović K., Filipić B., Kojić M., Filipović N., Stevanović M.M., Milenković M.T. (2022). In Vitro Colistin Susceptibility of Pandrug-Resistant Ac. Baumannii Is Restored in the Presence of Selenium Nanoparticles. J. Appl. Microbiol..

[23] Jovcic B., Novovic K., Dekic S., Hrenovic J. (2021). Colistin Resistance in Environmental Isolates of *Acinetobacter baumannii*. Microb. Drug Resist..

[24] Nurtop E., Bayındır Bilman F., Menekse S., Kurt Azap O., Gönen M., Ergonul O., Can F. (2019). Promoters of Colistin Resistance in *Acinetobacter baumannii* Infections. Microb. Drug Resist..

[25] Kline T., Trent M.S., Stead C.M., Lee M.S., Sousa M.C., Felise H.B., Nguyen H.V., Miller S.I. (2008). Synthesis of and Evaluation of Lipid A Modification by 4-Substituted 4-Deoxy Arabinose Analogs as Potential Inhibitors of Bacterial Polymyxin Resistance. Bioorganic Med. Chem. Lett..

[26] Nowak J., Zander E., Stefanik D., Higgins P.G., Roca I., Vila J., McConnell M.J., Cisneros J.M., Seifert H. (2017). MagicBullet Working Group WP4 High Incidence of Pandrug-Resistant *Acinetobacter baumannii* Isolates Collected from Patients with Ventilator-Associated Pneumonia in Greece, Italy and Spain as Part of the MagicBullet Clinical Trial. J. Antimicrob. Chemother..

[27] Olaitan A.O., Morand S., Rolain J.-M. (2014). Mechanisms of Polymyxin Resistance: Acquired and Intrinsic Resistance in Bacteria. Front. Microbiol..

[28] Chin C.-Y., Gregg K.A., Napier B.A., Ernst R.K., Weiss D.S. (2015). A PmrB-Regulated Deacetylase Required for Lipid A Modification and Polymyxin Resistance in *Acinetobacter baumannii*. Antimicrob. Agents Chemother..

[29] Gerson S., Betts J.W., Lucaßen K., Nodari C.S., Wille J., Josten M., Göttig S., Nowak J., Stefanik D., Roca I. (2019). Investigation of Novel *pmrB* and *eptA* Mutations in Isogenic *Acinetobacter baumannii* Isolates Associated with Colistin Resistance and Increased Virulence In Vivo. Antimicrob. Agents Chemother..

[30] Qureshi Z.A., Hittle L.E., O’Hara J.A., Rivera J.I., Syed A., Shields R.K., Pasculle A.W., Ernst R.K., Doi Y. (2015). Colistin-Resistant *Acinetobacter baumannii*: Beyond Carbapenem Resistance. Clin. Infect. Dis..

[31] Pelletier M.R., Casella L.G., Jones J.W., Adams M.D., Zurawski D.V., Hazlett K.R.O., Doi Y., Ernst R.K. (2013). Unique Structural Modifications Are Present in the Lipopolysaccharide from Colistin-Resistant Strains of *Acinetobacter baumannii*. Antimicrob. Agents Chemother..

[32] Arroyo L.A., Herrera C.M., Fernandez L., Hankins J.V., Trent M.S., Hancock R.E.W. (2011). The *pmrCAB* Operon Mediates Polymyxin Resistance in *Acinetobacter baumannii* ATCC 17978 and Clinical Isolates through Phosphoethanolamine Modification of Lipid A. Antimicrob. Agents Chemother..

[33] Beceiro A., Llobet E., Aranda J., Bengoechea J.A., Doumith M., Hornsey M., Dhanji H., Chart H., Bou G., Livermore D.M. (2011). Phosphoethanolamine Modification of Lipid A in Colistin-Resistant Variants of *Acinetobacter baumannii* Mediated by the pmrAB Two-Component Regulatory System. Antimicrob. Agents Chemother..

[34] Karakonstantis S. (2021). A Systematic Review of Implications, Mechanisms, and Stability of in Vivo Emergent Resistance to Colistin and Tigecycline in *Acinetobacter baumannii*. J. Chemother..

[35] Haeili M., Kafshdouz M., Feizabadi M.M. (2018). Molecular Mechanisms of Colistin Resistance Among Pandrug-Resistant Isolates of *Acinetobacter baumannii* with High Case-Fatality Rate in Intensive Care Unit Patients. Microb. Drug Resist..

[36] Khoshnood S., Savari M., Abbasi Montazeri E., Farajzadeh Sheikh A. (2020). Survey on Genetic Diversity, Biofilm Formation, and Detection of Colistin Resistance Genes in Clinical Isolates of *Acinetobacter baumannii*. IDR.

[37] Park Y.K., Choi J.Y., Shin D., Ko K.S. (2011). Correlation between Overexpression and Amino Acid Substitution of the PmrAB Locus and Colistin Resistance in *Acinetobacter baumannii*. Int. J. Antimicrob. Agents.

[38] Leite G.C., Stabler R.A., Neves P., Perdigão Neto L.V., Ruedas Martins R.C., Rizek C., Rossi F., Levin A.S., Costa S.F. (2019). Genetic and Virulence Characterization of Colistin-Resistant and Colistin-Sensitive A. Baumannii Clinical Isolates. Diagn. Microbiol. Infect. Dis..

[39] Kabic J., Novovic K., Kekic D., Trudic A., Opavski N., Dimkic I., Jovcic B., Gajic I. (2023). Comparative Genomics and Molecular Epidemiology of Colistin-Resistant *Acinetobacter baumannii*. Comput. Struct. Biotechnol. J..

[40] Trebosc V., Gartenmann S., Tötzl M., Lucchini V., Schellhorn B., Pieren M., Lociuro S., Gitzinger M., Tigges M., Bumann D. (2019). Dissecting Colistin Resistance Mechanisms in Extensively Drug-Resistant *Acinetobacter baumannii* Clinical Isolates. mBio.

[41] Deveson Lucas D., Crane B., Wright A., Han M.-L., Moffatt J., Bulach D., Gladman S.L., Powell D., Aranda J., Seemann T. (2018). Emergence of High-Level Colistin Resistance in an *Acinetobacter baumannii* Clinical Isolate Mediated by Inactivation of the Global Regulator H-NS. Antimicrob. Agents Chemother..

[42] Ngbede E.O., Poudel A., Kalalah A., Yang Y., Adekanmbi F., Adikwu A.A., Adamu A.M., Mamfe L.M., Daniel S.T., Useh N.M. (2020). Identification of Mobile Colistin Resistance Genes (Mcr-1.1, Mcr-5 and Mcr-8.1) in Enterobacteriaceae and Alcaligenes Faecalis of Human and Animal Origin, Nigeria. Int. J. Antimicrob. Agents.

[43] Andrade F.F., Silva D., Rodrigues A., Pina-Vaz C. (2020). Colistin Update on Its Mechanism of Action and Resistance, Present and Future Challenges. Microorganisms.

[44] Khuntayaporn P., Thirapanmethee K., Chomnawang M.T. (2022). An Update of Mobile Colistin Resistance in Non-Fermentative Gram-Negative Bacilli. Front. Cell. Infect. Microbiol..

[45] Martins-Sorenson N., Snesrud E., Xavier D.E., Cacci L.C., Iavarone A.T., McGann P., Riley L.W., Moreira B.M. (2020). A Novel Plasmid-Encoded Mcr-4.3 Gene in a Colistin-Resistant *Acinetobacter baumannii* Clinical Strain. J. Antimicrob. Chemother..

[46] Hameed F., Khan M.A., Muhammad H., Sarwar T., Bilal H., Rehman T.U. (2019). Plasmid-Mediated Mcr-1 Gene in *Acinetobacter baumannii* and Pseudomonas Aeruginosa: First Report from Pakistan. Rev. Soc. Bras. Med. Trop..

[47] Vila J., Martí S., Sánchez-Céspedes J. (2007). Porins, Efflux Pumps and Multidrug Resistance in *Acinetobacter baumannii*. J. Antimicrob. Chemother..

[48] Lee K., Yong D., Jeong S.H., Chong Y. (2011). Multidrug-Resistant *Acinetobacter* spp.: Increasingly Problematic Nosocomial Pathogens. Yonsei Med. J..

[49] Hood M.I., Becker K.W., Roux C.M., Dunman P.M., Skaar E.P. (2013). Genetic Determinants of Intrinsic Colistin Tolerance in *Acinetobacter baumannii*. Infect. Immun..

[50] Lean S.-S., Suhaili Z., Ismail S., Rahman N.I.A., Othman N., Abdullah F.H., Jusoh Z., Yeo C.C., Thong K.-L. (2014). Prevalence and Genetic Characterization of Carbapenem- and Polymyxin-Resistant *Acinetobacter baumannii* Isolated from a Tertiary Hospital in Terengganu, Malaysia. ISRN Microbiol..

[51] Thi Khanh Nhu N., Riordan D.W., Do Hoang Nhu T., Thanh D.P., Thwaites G., Huong Lan N.P., Wren B.W., Baker S., Stabler R.A. (2016). The Induction and Identification of Novel Colistin Resistance Mutations in *Acinetobacter baumannii* and Their Implications. Sci. Rep..

[52] El-Halfawy O.M., Valvano M.A. (2015). Antimicrobial Heteroresistance: An Emerging Field in Need of Clarity. Clin. Microbiol. Rev..

[53] Li J., Rayner C.R., Nation R.L., Owen R.J., Spelman D., Tan K.E., Liolios L. (2006). Heteroresistance to Colistin in Multidrug-Resistant *Acinetobacter baumannii*. Antimicrob. Agents Chemother..

[54] Hawley J.S., Murray C.K., Jorgensen J.H. (2008). Colistin Heteroresistance in *Acinetobacter* and Its Association with Previous Colistin Therapy. Antimicrob. Agents Chemother..

[55] Li J., Rayner C.R., Nation R.L., Deans R., Boots R., Widdecombe N., Douglas A., Lipman J. (2005). Pharmacokinetics of Colistin Methanesulfonate and Colistin in a Critically Ill Patient Receiving Continuous Venovenous Hemodiafiltration. Antimicrob. Agents Chemother..

[56] Tamma P.D., Aitken S.L., Bonomo R.A., Mathers A.J., van Duin D., Clancy C.J. (2023). Infectious Diseases Society of America 2023 Guidance on the Treatment of Antimicrobial Resistant Gram-Negative Infections. Clin. Infect Dis..

[57] Shields R.K., Paterson D.L., Tamma P.D. (2023). Navigating Available Treatment Options for Carbapenem-Resistant *Acinetobacter baumannii-Calcoaceticus* Complex Infections. Clin. Infect. Dis..

[58] Onorato L., De Luca I., Monari C., Coppola N. (2024). Cefiderocol Either in Monotherapy or Combination versus Best Available Therapy in the Treatment of Carbapenem-Resistant *Acinetobacter baumannii* Infections: A Systematic Review and Meta-Analysis. J. Infect..

[59] Gill K., Takamichi B., Cooper A. (2024). Clinical Efficacy of Cefiderocol-Based Regimens in Patients with Carbapenem-Resistant *Acinetobacter baumannii* Infections: New Data from CREDIBLE-CR with an Updated Meta-Analysis. Int. J. Antimicrob. Agents.

[60] Lyu C., Zhang Y., Liu X., Wu J., Zhang J. (2020). Clinical Efficacy and Safety of Polymyxins Based versus Non-Polymyxins Based Therapies in the Infections Caused by Carbapenem-Resistant *Acinetobacter baumannii*: A Systematic Review and Meta-Analysis. BMC Infect. Dis..

[61] Mei H., Yang T., Wang J., Wang R., Cai Y. (2019). Efficacy and Safety of Tigecycline in Treatment of Pneumonia Caused by MDR *Acinetobacter baumannii*: A Systematic Review and Meta-Analysis. J. Antimicrob. Chemother..

[62] Liu J., Shu Y., Zhu F., Feng B., Zhang Z., Liu L., Wang G. (2021). Comparative Efficacy and Safety of Combination Therapy with High-Dose Sulbactam or Colistin with Additional Antibacterial Agents for Multiple Drug-Resistant and Extensively Drug-Resistant *Acinetobacter baumannii* Infections: A Systematic Review and Network Meta-Analysis. J. Glob. Antimicrob. Resist..

[63] Kengkla K., Kongpakwattana K., Saokaew S., Apisarnthanarak A., Chaiyakunapruk N. (2018). Comparative Efficacy and Safety of Treatment Options for MDR and XDR *Acinetobacter baumannii* Infections: A Systematic Review and Network Meta-Analysis. J. Antimicrob. Chemother..

[64] Jung S.Y., Lee S.H., Lee S.Y., Yang S., Noh H., Chung E.K., Lee J.I. (2017). Antimicrobials for the Treatment of Drug-Resistant *Acinetobacter baumannii* Pneumonia in Critically Ill Patients: A Systemic Review and Bayesian Network Meta-Analysis. Crit. Care.

[65] Paul M., Carrara E., Retamar P., Tängdén T., Bitterman R., Bonomo R.A., De Waele J., Daikos G.L., Akova M., Harbarth S. (2022). European Society of Clinical Microbiology and Infectious Diseases (ESCMID) Guidelines for the Treatment of Infections Caused by Multidrug-Resistant Gram-Negative Bacilli (Endorsed by European Society of Intensive Care Medicine). Clin. Microbiol. Infect..

[66] Karakonstantis S., Rousaki M., Vassilopoulou L., Kritsotakis E.I. (2024). Global Prevalence of Cefiderocol Non-Susceptibility in Enterobacterales, Pseudomonas Aeruginosa, *Acinetobacter baumannii*, and Stenotrophomonas Maltophilia: A Systematic Review and Meta-Analysis. Clin. Microbiol. Infect..

[67] Karakonstantis S., Rousaki M., Kritsotakis E.I. (2022). Cefiderocol: Systematic Review of Mechanisms of Resistance, Heteroresistance and In Vivo Emergence of Resistance. Antibiotics.

[68] Martins-Gonçalves T., Pimenta J.S., Fontana H., Esposito F., Melocco G., Dantas K., Vásquez-Ponce F., Carrara F.E., Vespero E.C., Lincopan N. (2024). *Acinetobacter baumannii* International Clone 2 Co-Producing OXA-23, NDM-1, and ArmA Emerging in South America. Antimicrob. Agents Chemother..

[69] Tsilipounidaki K., Gkountinoudis C.-G., Florou Z., Fthenakis G.C., Miriagou V., Petinaki E. (2023). The Molecular Characterization of blaNDM-1-Positive *Acinetobacter baumannii* Isolated in Central Greece. Microorganisms.

[70] Miller A.A., Moussa S.H., McLeod S.M. (2024). Characterization of *Acinetobacter baumannii-Calcoaceticus* Complex Isolates and Microbiological Outcome for Patients Treated with Sulbactam-Durlobactam in a Phase 3 Trial (ATTACK). Antimicrob. Agents Chemother..

[71] Iovleva A., McElheny C.L., Fowler E.L., Cober E., Herc E.S., Arias C.A., Hill C., Baum K., Fowler V.G., Chambers H.F. (2024). In Vitro Activity of Sulbactam-Durlobactam against Colistin-Resistant and/or Cefiderocol-Non-Susceptible, Carbapenem-Resistant *Acinetobacter baumannii* Collected in U.S. Hospitals. Antimicrob. Agents Chemother..

[72] Kaye K.S., Shorr A.F., Wunderink R.G., Du B., Poirier G.E., Rana K., Miller A., Lewis D., O’Donnell J., Chen L. (2023). Efficacy and Safety of Sulbactam–Durlobactam versus Colistin for the Treatment of Patients with Serious Infections Caused by *Acinetobacter baumannii*–Calcoaceticus Complex: A Multicentre, Randomised, Active-Controlled, Phase 3, Non-Inferiority Clinical Trial (ATTACK). Lancet Infect. Dis..

[73] Bassetti M., Echols R., Matsunaga Y., Ariyasu M., Doi Y., Ferrer R., Lodise T.P., Naas T., Niki Y., Paterson D.L. (2021). Efficacy and Safety of Cefiderocol or Best Available Therapy for the Treatment of Serious Infections Caused by Carbapenem-Resistant Gram-Negative Bacteria (CREDIBLE-CR): A Randomised, Open-Label, Multicentre, Pathogen-Focused, Descriptive, Phase 3 Trial. Lancet Infect. Dis..

[74] Wunderink R.G., Matsunaga Y., Ariyasu M., Clevenbergh P., Echols R., Kaye K.S., Kollef M., Menon A., Pogue J.M., Shorr A.F. (2021). Cefiderocol versus High-Dose, Extended-Infusion Meropenem for the Treatment of Gram-Negative Nosocomial Pneumonia (APEKS-NP): A Randomised, Double-Blind, Phase 3, Non-Inferiority Trial. Lancet Infect. Dis..

[75] Petropoulou D., Siopi M., Vourli S., Pournaras S. (2022). Activity of Sulbactam-Durlobactam and Comparators Against a National Collection of Carbapenem-Resistant *Acinetobacter baumannii* Isolates From Greece. Front. Cell. Infect. Microbiol..

[76] Jaruratanasirikul S., Nitchot W., Wongpoowarak W., Samaeng M., Nawakitrangsan M. (2019). Population Pharmacokinetics and Monte Carlo Simulations of Sulbactam to Optimize Dosage Regimens in Patients with Ventilator-Associated Pneumonia Caused by *Acinetobacter baumannii*. Eur. J. Pharm. Sci..

[77] Tsuji B.T., Pogue J.M., Zavascki A.P., Paul M., Daikos G.L., Forrest A., Giacobbe D.R., Viscoli C., Giamarellou H., Karaiskos I. (2019). International Consensus Guidelines for the Optimal Use of the Polymyxins: Endorsed by the American College of Clinical Pharmacy (ACCP), European Society of Clinical Microbiology and Infectious Diseases (ESCMID), Infectious Diseases Society of America (IDSA), International Society for Anti-infective Pharmacology (ISAP), Society of Critical Care Medicine (SCCM), and Society of Infectious Diseases Pharmacists (SIDP). Pharmacotherapy.

[78] Karaiskos I., Gkoufa A., Polyzou E., Schinas G., Athanassa Z., Akinosoglou K. (2023). High-Dose Nebulized Colistin Methanesulfonate and the Role in Hospital-Acquired Pneumonia Caused by Gram-Negative Bacteria with Difficult-to-Treat Resistance: A Review. Microorganisms.

[79] Andrianopoulos I., Kazakos N., Lagos N., Maniatopoulou T., Papathanasiou A., Papathanakos G., Koulenti D., Toli E., Gartzonika K., Koulouras V. (2024). Co-Administration of High-Dose Nebulized Colistin for *Acinetobacter baumannii* Bacteremic Ventilator-Associated Pneumonia: Impact on Outcomes. Antibiotics.

[80] Feng J.-Y., Huang J.-R., Lee C.-C., Tseng Y.-H., Pan S.-W., Chen Y.-M., Yang K.-Y. (2023). Role of Nebulized Colistin as a Substitutive Strategy against Nosocomial Pneumonia Caused by CR-GNB in Intensive Care Units: A Retrospective Cohort Study. Ann. Intensive Care.

[81] Sodeifian F., Zangiabadian M., Arabpour E., Kian N., Yazarlou F., Goudarzi M., Centis R., Seghatoleslami Z.S., Kameh M.C., Danaei B. (2023). Tigecycline-Containing Regimens and Multi Drug-Resistant *Acinetobacter baumannii*: A Systematic Review and Meta-Analysis. Microb. Drug Resist..

[82] Tsakris A., Koumaki V., Dokoumetzidis A. (2019). Minocycline Susceptibility Breakpoints for *Acinetobacter baumannii*: Do We Need to Re-Evaluate Them?. J. Antimicrob. Chemother..

[83] Scott C.J., Zhu E., Jayakumar R.A., Shan G., Viswesh V. (2022). Efficacy of Eravacycline *Versus* Best Previously Available Therapy for Adults With Pneumonia Due to Difficult-to-Treat Resistant (DTR) *Acinetobacter baumannii*. Ann. Pharmacother..

[84] Alexander C., Hill D. (2024). A Retrospective Case-Control Study of Eravacycline for the Treatment of Carbapenem-Resistant Acinetobacter Infections in Patients With Burn Injuries. J. Burn. Care Res..

[85] Jackson M.N.W., Wei W., Mang N.S., Prokesch B.C., Ortwine J.K. (2024). Combination Eravacycline Therapy for Ventilator-associated Pneumonia Due to Carbapenem-resistant *Acinetobacter baumannii* in Patients with COVID-19: A Case Series. Pharmacotherapy.

[86] Buckley V., Tran M., Price T., Singh S., Stramel S. (2023). Use of Eravacycline for *Acinetobacter baumannii* Infections: A Case Series. J. Pharm. Pract..

[87] Guastalegname M., Trecarichi E.M., Russo A. (2023). Intravenous Fosfomycin: The Underdog Player in the Treatment of Carbapenem-Resistant *Acinetobacter baumannii* Infections. Clin. Infect. Dis..

[88] Sirijatuphat R., Thamlikitkul V. (2014). Preliminary Study of Colistin versus Colistin plus Fosfomycin for Treatment of Carbapenem-Resistant *Acinetobacter baumannii* Infections. Antimicrob. Agents Chemother..

[89] Assimakopoulos S.F., Karamouzos V., Eleftheriotis G., Lagadinou M., Bartzavali C., Kolonitsiou F., Paliogianni F., Fligou F., Marangos M. (2023). Efficacy of Fosfomycin-Containing Regimens for Treatment of Bacteremia Due to Pan-Drug Resistant *Acinetobacter baumannii* in Critically Ill Patients: A Case Series Study. Pathogens.

[90] Russo A., Bassetti M., Bellelli V., Bianchi L., Marincola Cattaneo F., Mazzocchetti S., Paciacconi E., Cottini F., Schiattarella A., Tufaro G. (2021). Efficacy of a Fosfomycin-Containing Regimen for Treatment of Severe Pneumonia Caused by Multidrug-Resistant *Acinetobacter baumannii*: A Prospective, Observational Study. Infect. Dis. Ther..

[91] Pasteran F., Cedano J., Baez M., Albornoz E., Rapoport M., Osteria J., Montaña S., Le C., Ra G., Bonomo R.A. (2021). A New Twist: The Combination of Sulbactam/Avibactam Enhances Sulbactam Activity against Carbapenem-Resistant *Acinetobacter baumannii* (CRAB) Isolates. Antibiotics.

[92] Rodriguez C.H., Brune A., Nastro M., Vay C., Famiglietti A. (2020). In Vitro Synergistic Activity of the Sulbactam/Avibactam Combination against Extensively Drug-Resistant *Acinetobacter baumannii*. J. Med. Microbiol..

[93] Falagas M.E., Vardakas K.Z., Roussos N.S. (2015). Trimethoprim/Sulfamethoxazole for Acinetobacter Spp.: A Review of Current Microbiological and Clinical Evidence. Int. J. Antimicrob. Agents.

[94] Raz-Pasteur A., Liron Y., Amir-Ronen R., Abdelgani S., Ohanyan A., Geffen Y., Paul M. (2019). Trimethoprim-Sulfamethoxazole vs. Colistin or Ampicillin–Sulbactam for the Treatment of Carbapenem-Resistant *Acinetobacter baumannii*: A Retrospective Matched Cohort Study. J. Glob. Antimicrob. Resist..

[95] Luna B., Trebosc V., Lee B., Bakowski M., Ulhaq A., Yan J., Lu P., Cheng J., Nielsen T., Lim J. (2020). A Nutrient-Limited Screen Unmasks Rifabutin Hyperactivity for Extensively Drug-Resistant *Acinetobacter baumannii*. Nat. Microbiol..

[96] Trebosc V., Schellhorn B., Schill J., Lucchini V., Bühler J., Bourotte M., Butcher J.J., Gitzinger M., Lociuro S., Kemmer C. (2020). In Vitro Activity of Rifabutin against 293 Contemporary Carbapenem-Resistant *Acinetobacter baumannii* Clinical Isolates and Characterization of Rifabutin Mode of Action and Resistance Mechanisms. J. Antimicrob. Chemother..

[97] Cheng J., Yan J., Reyna Z., Slarve M., Lu P., Spellberg B., Luna B. (2021). Synergistic Rifabutin and Colistin Reduce Emergence of Resistance When Treating *Acinetobacter baumannii*. Antimicrob. Agents Chemother..

[98] Antraygues K., Maingot M., Schellhorn B., Trebosc V., Gitzinger M., Deprez B., Defert O., Dale G.E., Bourotte M., Lociuro S. (2022). Design and Synthesis of Water-Soluble Prodrugs of Rifabutin for Intraveneous Administration. Eur. J. Med. Chem..

[99] Trebosc V., Kemmer C., Lociuro S., Gitzinger M., Dale G.E. (2021). Rifabutin for Infusion (BV100) for the Treatment of Severe Carbapenem-Resistant *Acinetobacter baumannii* Infections. Drug Discov. Today.

[100] Phillips M.C., Wald-Dickler N., Loomis K., Luna B.M., Spellberg B. (2020). Pharmacology, Dosing, and Side Effects of Rifabutin as a Possible Therapy for Antibiotic-Resistant Acinetobacter Infections. Open Forum Infect. Dis..

[101] Miller S., Goy K., She R., Spellberg B., Luna B. (2023). Antimicrobial Susceptibility Testing Performed in RPMI 1640 Reveals Azithromycin Efficacy against Carbapenem-Resistant *Acinetobacter baumannii* and Predicts In Vivo Outcomes in Galleria Mellonella. Antimicrob. Agents Chemother..

[102] Lin L., Nonejuie P., Munguia J., Hollands A., Olson J., Dam Q., Kumaraswamy M., Rivera H., Corriden R., Rohde M. (2015). Azithromycin Synergizes with Cationic Antimicrobial Peptides to Exert Bactericidal and Therapeutic Activity Against Highly Multidrug-Resistant Gram-Negative Bacterial Pathogens. EBioMedicine.

[103] Dillon N., Holland M., Tsunemoto H., Hancock B., Cornax I., Pogliano J., Sakoulas G., Nizet V. (2019). Surprising Synergy of Dual Translation Inhibition vs. *Acinetobacter baumannii* and Other Multidrug-Resistant Bacterial Pathogens. eBioMedicine.

[104] Karakonstantis S. (2022). Re: ‘Macrolide Therapy in Pseudomonas Aeruginosa Infections Causes uL4 Ribosomal Protein Mutations Leading to High-Level Resistance’ by Goltermann et al. Clin. Microbiol. Infect..

[105] Karakonstantis S., Ioannou P., Kofteridis D.D. (2022). In Search for a Synergistic Combination against Pandrug-Resistant A. Baumannii; Methodological Considerations. Infection.

[106] Maraolo A.E., Ong D.S.Y. (2023). Colistin plus Meropenem versus Colistin Alone for Invasive Infections Caused by Carbapenem-Resistant *Acinetobacter baumannii*: A Rapid Systematic Review of Randomized Controlled Trials Using Bayesian Meta-Analysis. Clin. Microbiol. Infect..

[107] Huang C., Chen I., Tang T. (2022). Colistin Monotherapy versus Colistin plus Meropenem Combination Therapy for the Treatment of Multidrug-Resistant *Acinetobacter baumannii* Infection: A Meta-Analysis. JCM.

[108] Park H.J., Cho J.H., Kim H.J., Han S.H., Jeong S.H., Byun M.K. (2019). Colistin Monotherapy versus Colistin/Rifampicin Combination Therapy in Pneumonia Caused by Colistin-Resistant *Acinetobacter baumannii*: A Randomised Controlled Trial. J. Glob. Antimicrob. Resist..

[109] Durante-Mangoni E., Signoriello G., Andini R., Mattei A., De Cristoforo M., Murino P., Bassetti M., Malacarne P., Petrosillo N., Galdieri N. (2013). Colistin and Rifampicin Compared With Colistin Alone for the Treatment of Serious Infections Due to Extensively Drug-Resistant *Acinetobacter baumannii*: A Multicenter, Randomized Clinical Trial. Clin. Infect. Dis..

[110] Aydemir H., Akduman D., Piskin N., Comert F., Horuz E., Terzi A., Kokturk F., Ornek T., Celebi G. (2013). Colistin *vs*. the Combination of Colistin and Rifampicin for the Treatment of Carbapenem-Resistant *Acinetobacter baumannii* Ventilator-Associated Pneumonia. Epidemiol. Infect..

[111] Karakonstantis S., Kritsotakis E.I. (2021). Systematic Review and Meta-Analysis of the Proportion and Associated Mortality of Polymicrobial (vs Monomicrobial) Pulmonary and Bloodstream Infections by *Acinetobacter baumannii* Complex. Infection.

[112] Mohd Sazlly Lim S., Heffernan A.J., Roberts J.A., Sime F.B. (2021). Semimechanistic Pharmacokinetic/Pharmacodynamic Modeling of Fosfomycin and Sulbactam Combination against Carbapenem-Resistant *Acinetobacter baumannii*. Antimicrob. Agents Chemother..

[113] Mohd Sazlly Lim S., Heffernan A.J., Zowawi H.M., Roberts J.A., Sime F.B. (2021). Semi-Mechanistic PK/PD Modelling of Meropenem and Sulbactam Combination against Carbapenem-Resistant Strains of *Acinetobacter baumannii*. Eur. J. Clin. Microbiol. Infect. Dis..

[114] Mohd Sazlly Lim S., Heffernan A.J., Roberts J.A., Sime F.B. (2021). Pharmacodynamic Analysis of Meropenem and Fosfomycin Combination Against Carbapenem-Resistant *Acinetobacter baumannii* in Patients with Normal Renal Clearance: Can It Be a Treatment Option?. Microb. Drug Resist..

[115] Mohd Sazlly Lim S., Heffernan A., Naicker S., Wallis S., Roberts J.A., Sime F.B. (2022). Evaluation of Fosfomycin-Sulbactam Combination Therapy against Carbapenem-Resistant *Acinetobacter baumannii* Isolates in a Hollow-Fibre Infection Model. Antibiotics.

